# Differential Effects of Reperfusion on Cardiac Mitochondrial Subpopulations in a Preclinical Porcine Model of Acute Myocardial Infarction

**DOI:** 10.3389/fcell.2022.843733

**Published:** 2022-03-09

**Authors:** Kadambari Chandra Shekar, Demetris Yannopoulos, Marinos Kosmopoulos, Matthias L. Riess

**Affiliations:** ^1^ Integrative Biology and Physiology, University of Minnesota at Twin Cities, St. Paul, MN, United States; ^2^ Department of Cardiology, Division of Medicine, University of Minnesota at Twin Cities, St. Paul, MN, United States; ^3^ Anesthesiology, TVHS VA Medical Center, Nashville, TN, United States; ^4^ Department of Anesthesiology, Vanderbilt University Medical Center, Nashville, TN, United States; ^5^ Department of Pharmacology, Vanderbilt University, Nashville, TN, United States

**Keywords:** mitochondria, reperfusion injury, preclinical, cardiovascular, SSM, IFM

## Abstract

Acute myocardial infarction (AMI) leads to localized cardiac ischemia and can be fatal if untreated. Despite being treatable, the threat of ischemia-reperfusion (IR) injury remains high. Mitochondria are central to both propagation and mitigation of IR injury, and cardiac mitochondria are categorized into two major subtypes—subsarcolemmal and interfibrillar mitochondria (SSM and IFM, respectively). We hypothesized that, in our pre-clinical porcine model of AMI, SSM and IFM are differentially affected by reperfusion. AMI was induced in female pigs by balloon occlusion of the left anterior descending artery for 45 min, followed by 4 h of reperfusion. At the end of reperfusion, animals were euthanized. Cardiac SSM and IFM from the affected ischemic area and a nearby non-ischemic area were isolated to compare mitochondrial function using substrates targeting mitochondrial electron transport chain complexes I and II. Despite detecting overall significant differences in mitochondrial function including yield, mitochondrial S3 and S4 respirations, and calcium retention, consistent individual functional differences in the two mitochondrial subpopulations were not observed, both between the two mitochondrial subtypes, as well as between the ischemic and non-ischemic tissue. Nonetheless, this study describes the mitochondrial subtype response within the initial few hours of reperfusion in a clinically relevant model of AMI, which provides valuable information needed to develop novel mitochondrially targeted therapies for AMI.

## 1 Introduction

Cardiovascular disease is the leading cause of death in the United States and worldwide. Americans experience acute myocardial infarctions (AMIs) at a rate of 1 every 40 s (Benjamin et al., 2019; Virani et al., 2020). AMI occurs when there is a sudden cessation of blood flow to a region of the heart muscle due to partial or complete occlusion of a major coronary artery. If left untreated, the ischemic area becomes permanently necrotic and develops scar tissue (de Zwaan et al., 2001). AMI greatly increases the risk of other adverse cardiac events including arrhythmia, heart failure, cardiac arrest and death (Zaman and Kovoor, 2014). Current treatments for AMI include percutaneous coronary interventions (PCI) using balloon angioplasty, and when not available, the use of thrombolytics to dissolve blood clots and re-establish blood flow (Arntz, 2008; Armstrong et al., 2013; O’Gara et al., 2013; Vogel et al., 2019). A particularly severe form of AMI, ST-elevation myocardial infarction (STEMI), is caused when the coronary artery is completely occluded, leading to irreversible necrosis of the affected tissue. As the acronym suggests, STEMI is characterized by elevation in the ST-segment in the electrocardiogram (ECG) (Ibanez et al., 2018).

In cardiomyocytes, loss of oxygen and substrates with ischemia leads to an inhibition of the Krebs cycle, reduced ATP production, increased reactive oxygen species (ROS) generation, accumulation of intracellular Na^+^ and Ca^2+^ ions, and hence loss of myocyte contractility (Dhalla and Duhamel, 2007; Kalogeris et al., 2012), while reperfusion further increases Ca^2+^ overload and ROS accumulation. In the mitochondria, Ca^2+^ overload and ROS lead to the opening of the membrane permeability transition pore (mPTP), resulting in cell death (Lesnefsky et al., 2001). Hence, mitochondria make attractive targets to treat reperfusion injury.

Cardiac mitochondria exists as two major subpopulations—subsarcolemmal mitochondria (SSM) and interfibrillar mitochondria (IFM). Originally described as being different based on the spatial location (Palmer et al., 1977), over the past 70 years, several studies have extensively documented the differences between SSM and IFM in terms of structure, function, location and role in pathophysiological models (Palmer et al., 1977, 1985; Shekar et al., 2014). However, no studies thus far have explored the role of these two mitochondrial subtypes in a large animal model of AMI in the duration it takes for the infarction to develop. In this study, we sought to determine if the two cardiac mitochondrial subpopulations of the heart respond differently to the downstream effects of AMI. The goal of this study was to describe possible mitochondrial functional differences in the two cardiac mitochondrial subpopulations in a porcine model of AMI reperfusion. We hypothesized that both the SSM and IFM have differentially altered functions in the ischemic region after AMI and tested this hypothesis using an established model of AMI induced by left anterior descending (LAD) artery occlusion, where the mitochondrial function of the ischemic region was compared to the non-ischemic region of the same heart.

## 2 Materials and Methods

The data that support the findings of this study are available from the corresponding author upon reasonable request.

### 2.1 Experimental Design

All animal studies were performed at the University of Minnesota Advance Pre-clinical Imaging Center according to the National Research Council’s Guidelines for the Care and Use of Laboratory Animals (8th edition) with the approval of the University of Minnesota Institutional Animal Care and Use Committee.

### 2.2 Animal Model

Ten female Yorkshire pigs, about 3-4 months old, weighing 47.0 ± 0.8 kg were used for the purpose of this study. The surgical preparation and anesthesia were previously described (Bartos et al., 2016). Briefly, the pigs were anesthetized using ketamine (20–30 mg kg^−1^, intramuscular) and xylazine (1–3 mg kg^−1^, intramuscular) for initial sedation. Intubation was performed with a 7.0 mm inner diameter endotracheal tube. After intubation, the remainder of the study was completed using inhaled isoflurane continuously at an end-tidal concentration of 0.8-1.5%. A volume-controlled ventilator (Narkomed 2A, Dräger, PA, United States) was set to a tidal volume of 10 ml kg^−1^, and the respiratory rate was adjusted to maintain the end tidal carbon dioxide concentration between 36–45 mmHg; F_i_O_2_ was kept at 30% to result in a PaO_2_ of at least 80 mmHg and an oxygen saturation above 90%. Cardiac electrical activity was measured using a 3-lead ECG. Hemodynamic parameters including systolic, diastolic pressures and right atrial pressures were continuously measured using Millar catheters (Millar Instruments, Houston, TX, United States) placed at the femoral artery and external right jugular vein, respectively, through an 8F vascular access sheath placed under ultrasound guidance. A right ear vein catheter was placed for additional intravenous access. Body temperature was measured using an esophageal temperature probe and maintained at 37.5 ± 0.5°C. Before the start of the PCI, all animals received a one-time bolus of 5,000 units of intravenous heparin and 1,000 units of heparin every 2 hours thereafter. All hemodynamic data were recorded and analyzed using LabView (v.2015, National Instruments, Austin TX, United States).

### 2.3 Experimental Protocol

**FIGURE 1:**
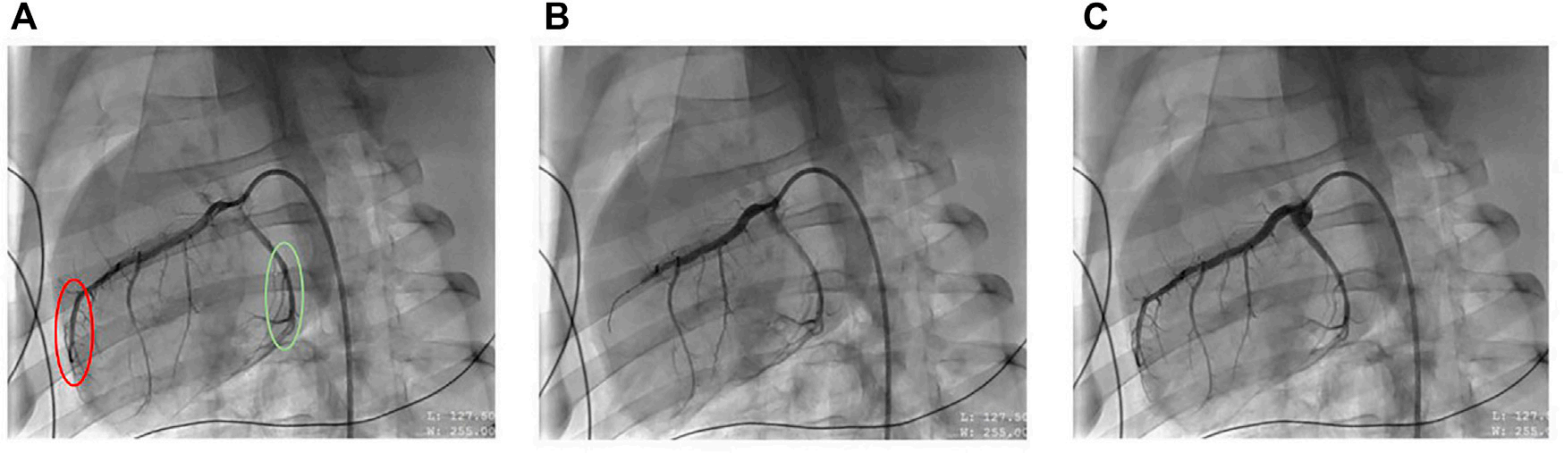
Representative X-ray fluoroscopic images of coronary artery blood flow before (left), during (middle) and after (right) mid-left anterior descending (LAD) artery occlusion, resulting in ST-elevation myocardial infarction (STEMI) **(A)** represents the porcine heart at baseline where the red circle denotes the ischemic LAD artery region, and the green circle denotes non-ischemic circumflex artery region. **(B)** represents the heart when the LAD is occluded, causing ischemia and **(C)** represents the heart after balloon deflation, resulting in reperfusion.

This model of STEMI was described previously (Bartos et al., 2016). STEMI was induced by first inserting a 0.014 in. guidewire into the LAD and advancing a 2.75 × 8 mm monorail balloon (Medtronic, Minneapolis, MN, United States) to the second diagonal branch under fluoroscopy (Figure 1A). The balloon was then inflated, and occlusion confirmed by injecting contrast for imaging fluoroscopy (Figure 1B). STEMI was further confirmed by ST-changes in the ECG. The occlusion period lasted for 45 min. Prior to balloon deflation, all animals received a one-time bolus of 40 mg intravenous amiodarone. Subsequently, the balloon was deflated, and reperfusion was once again confirmed by fluoroscopy to verify blood flow to the previously occluded artery (Figure 1C). During this time, any arrhythmia including ventricular fibrillation was treated with non-synchronized defibrillations (biphasic, 300 J) as needed. All animals that achieved return of spontaneous circulation (ROSC) within 15 min of the arrhythmic event were included in the study. This model achieves an infarct size of ∼40% relative to the area at risk (Bartos et al., 2016).

### 2.4 Cardiac Function and Injury Markers

At baseline before the start of the study and at 4 h of reperfusion, assessment of cardiac injury was performed by measuring the plasma levels of troponin I and Creatine Kinase-MB Isoform (CK-MB) from arterial blood using 2-site sandwich ELISA (Stratus CS Acute Care, Munich, Germany). Similarly, cardiac function was analyzed by determining the ejection fraction (EF%) along the ventricular short axis, at the above timepoints using echocardiography (Siemens Acuson X300, Philadelphia, PA, United States).

### 2.5 Tissue Harvest and Isolation of Cardiac Mitochondrial Subpopulations

At the end of the study (4 h from the start of reperfusion), anesthetized animals were euthanized by cardiac harvest. Cardiac tissue was obtained from two areas of the left ventricle—one from the LAD area immediately distal to the balloon placement and a second from an adjacent non-ischemic circumflex artery area unaffected by the STEMI. Cardiac SSM and IFM were isolated from *n* = 10 pigs using a well-established protocol (Palmer et al., 1977; Holmuhamedov et al., 2012; Shekar et al., 2014) with minor modifications. In short, ∼3 g of cardiac tissue from either the ischemic or the non-ischemic region was homogenized using 1:10 ice cold Chapel Perry buffer (100 mM KCl, 50 mM MOPS, 5 mM MgSO_4_, 1 mM EGTA, 1 mM ATP) using a combination of Polytron and Potter-Elvehjem homogenizers, followed by differential centrifugation at 580 x *g* for 10 min (twice). At this stage, the supernatants and the pellets were separated. SSM were released from the supernatant in the subsequent spins at 3,000 x *g*. To isolate IFM, the pellet from the previous step was treated with 5 mg g^−1^ wet weight (gww) trypsin for 10 min to release the interfibrillar mitochondria from the muscle fibers, followed by neutralization of trypsin using the isolation buffer containing Bovine Serum Albumin. Subsequently, this trypsin was removed by centrifugation at 7,500 x *g* and washed subsequently at 580 x *g* to remove excess trypsin. Finally, both the homogenates for SSM and IFM were centrifuged at 3,000 x *g,* resulting in final mitochondrial pellet suspended in KME (100 mM KCl, 50 mM MOPS, 0.5 mM EGTA) buffer*.* The final protein concentration was measured using Bradford (Bradford, 1976) assay (Sigma, St. Louis, MO, United States). Since the mitochondria obtained from this isolation protocol has been previously thoroughly characterized (Palmer et al., 1977, 1985; Holmuhamedov et al., 2012), no additional conformational testing was performed at the time of isolation. All mitochondrial experiments were normalized to mg protein.

### 2.6 Mitochondrial Function

#### 2.6.1 Mitochondrial Respiration

Mitochondrial respiration was measured at 25 °C using the Clarke electrode method (Strathkelvin instruments, North Lanarkshire, Scotland) and reported as previously described (Riess et al., 2014; Matsuura et al., 2017), About 0.5 mg ml^−1^ SSM or IFM were suspended in experimental buffer (130 mmol KCl, 5 mmol K_2_HPO_4_, 5 mmol MOPS, 0.1% BSA, pH adjusted to 7.15 with KOH) followed by the addition of either 10 mM final working concentration of pyruvate and malate (complex I substrates) or 10 µM final working concentration of rotenone followed by 10 mM final working concentration succinate (complex II substrate). Mitochondrial state 3 (S3) respiration was determined by measuring the chamber oxygen levels after adding 250 µM ADP. State 4 (S4) respiration was measured for 60 s after all the ADP was phosphorylated to ATP. The ratio of S3 and S4, computed as the respiratory control index (RCI) indicated the efficiency of the coupling of electron transport chain complexes to ATP production. For this assay, *n* = 7–10 animals per group were used. Variability in sample size was due to reduced mitochondrial yield in select samples and subsequently, limited availability of samples required for the assay.

#### 2.6.2 Calcium Uptake Assay

Mitochondrial tolerance to calcium stress was monitored using spectrophotometry and Ca Green-5N hexapotassium salt (Life technologies, Carlsbad, CA, United States) by measuring the total Ca^2+^ the mitochondria can hold before a sudden Ca^2+^ release by opening the mPTP (Riess et al., 2014; Matsuura et al., 2017). 0.5 mg ml^−1^ mitochondria were added to a cuvette containing calcium-free and phosphate-free experimental buffer and either complex I or complex II substrates. After 1 min of stabilization, a continuous infusion of 5 mM Ca^2+^ in the form of CaCl_2_ was started using a syringe pump at the rate of 30 μL min^−1^. The increase in extra-mitochondrial Ca^2+^ was measured at the excitation and emission wavelengths of 510 and 531 nm, respectively. Calcium retention capacity (CRC) was measured as the amount of Ca^2+^ infused until the opening of mPTP. The amount of Ca^2+^ corresponding to the time taken to reach maximum fluorescence values indicates the total CRC of the mitochondria. The higher the CRC, the higher the tolerance, the longer it takes for mPTP to open, and the more viable mitochondria are. For this assay, *n* = 9-10 animals per group were used. Variability in sample size was due to reduced mitochondrial yield in select samples, which subsequently limited availability of samples required for the assay.

#### 2.6.3 ATP Measurement by Luminometry

Mitochondrial rate of ATP production was measured using the ATP determination kit (Thermo Scientific ATP Determination Kit [A22066], Carlsbad, CA, United States) by the chemiluminescence method (Riess et al., 2014; Matsuura et al., 2017). Manufacturer’s instructions were followed to measure the mitochondrial ATP activity using a single tube luminometer (GloMax 20/20, Promega, Madison, WI, United States). For this assay, *n* = 7-8 animals were used. Variability in sample size was due to reduced mitochondrial yield in select samples combined with luminometer malfunction on two study days. All results were normalized to background ATP activity as measured with the addition of oligomycin to inhibit ATP synthase.

### 2.7 Statistical Analysis

For both cardiac function and mitochondrial function experiments, the Pratt test was used to determine if the data assume Gaussian distribution. Every datapoint collected from the study animals was included in the study. Since a majority of the outcomes followed non-gaussian distribution, all data are presented as median (interquartile range) and were analyzed using non-parametric methods. Wilcoxon matched pairs signed rank test was used to compare cardiac function at baseline and 4 h of reperfusion. Friedman’s test with Dunn’s pairwise comparison was used to compare the effect of injury in both mitochondrial subtypes and to determine mitochondrial differences between the two subpopulations within each region. *p* ≤ 0.05 (two-tailed) was considered significant. Prism 7.0 (GraphPad, San Diego, CA, United States) was used to analyze and graph the results.

## 3 Results

### 3.1 Myocardial Outcomes After STEMI

#### 3.1.1 Cardiac Function

Mean arterial pressure (MAP), echocardiography, and plasma biomarkers for cardiac damage (troponin I and CK-MB) were measured at baseline (pre-ischemia) and at 4 h of reperfusion for all animals. The median ejection fraction was at 60% (55, 60) at baseline and significantly reduced to 35% (23, 53) at 4 h of reperfusion. Similarly, the median MAPs significantly decreased from 86.3 (79.8, 95.8) mmHg to 60.8 (56.3, 66.2) mmHg. Combined, this confirms the reduction in cardiac function with reperfusion. Troponin I and CK-MB were also found to be significantly increased in plasma compared to baseline (Table 1).

**TABLE 1:** Comparison of cardiac function parameters at baseline and after 4 h of reperfusion. Results are represented as median (25th, 75th percentile) based on n = 10. *p* ≤ 0.05 (two-tailed) was considered significant.

Parameters	Baseline	4-h reperfusion	*p*-value
Mean arterial pressure (mmHg)	86.3 (79.8, 95.8)	60.8 (56.3, 66.2)	0.0059
Plasma Troponin I (µg L^−1^)	0.025 (0.008, 0.080)	148 (98.4, 163)	0.0020
Creatine kinase MB isoform (µg L^−1^)	3.00 (1.85, 3.50)	26.7 (20.9, 38.9)	0.0039
Ejection fraction (%)	60 (55, 60)	35 (23, 53)	0.0078

#### 3.1.2 Myocardial Infarction and Reperfusion Outcomes

Upon inducing STEMI (Figure 1B), any ventricular fibrillation observed was treated immediately with an average of two defibrillations. A total of two animals underwent defibrillations and achieved ROSC, and thus were included in the study. Of note, no obvious correlations were observed between the number of defibrillations and mitochondrial yield, and no additional statistical evaluation was performed due to the small sample size of the animals that received defibrillations.

### 3.2 Comparison of the Function of Cardiac Mitochondrial Subpopulations in AMI

**FIGURE 2:**
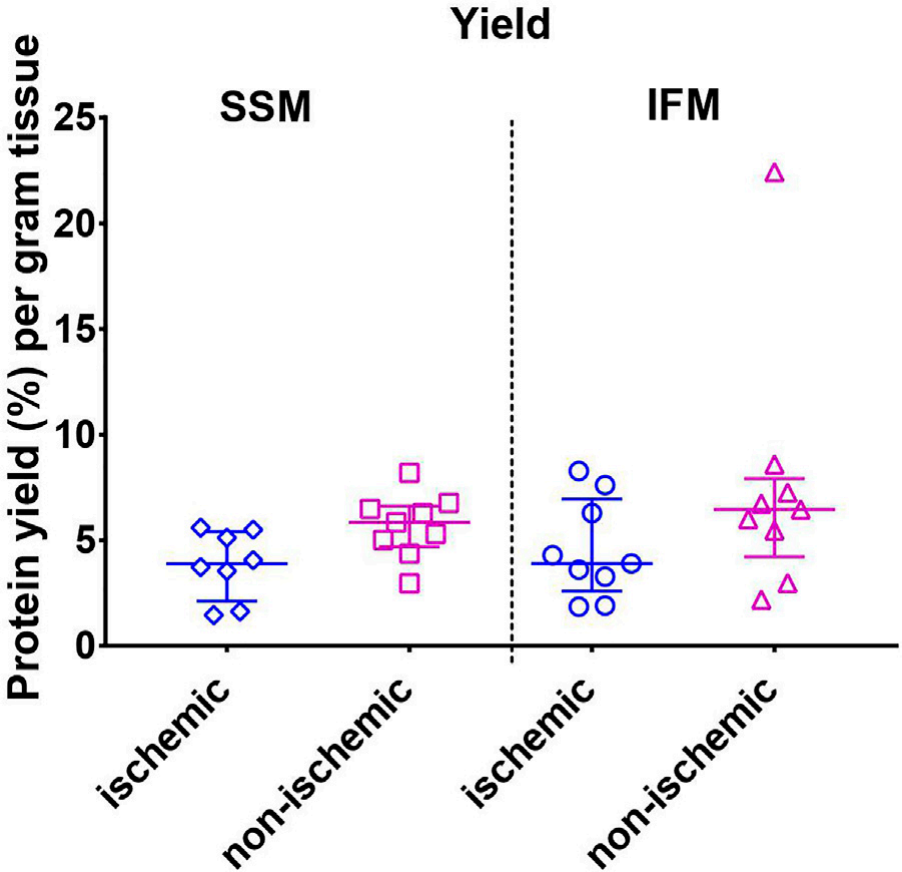
Mitochondrial protein yield as measured by the Bradford assay. Values are represented as median with interquartile range (*n* = 10/group). No significant differences were found among the four groups. For exact *p*-values of all group comparisons, please refer to Table 2.

**FIGURE 3:**
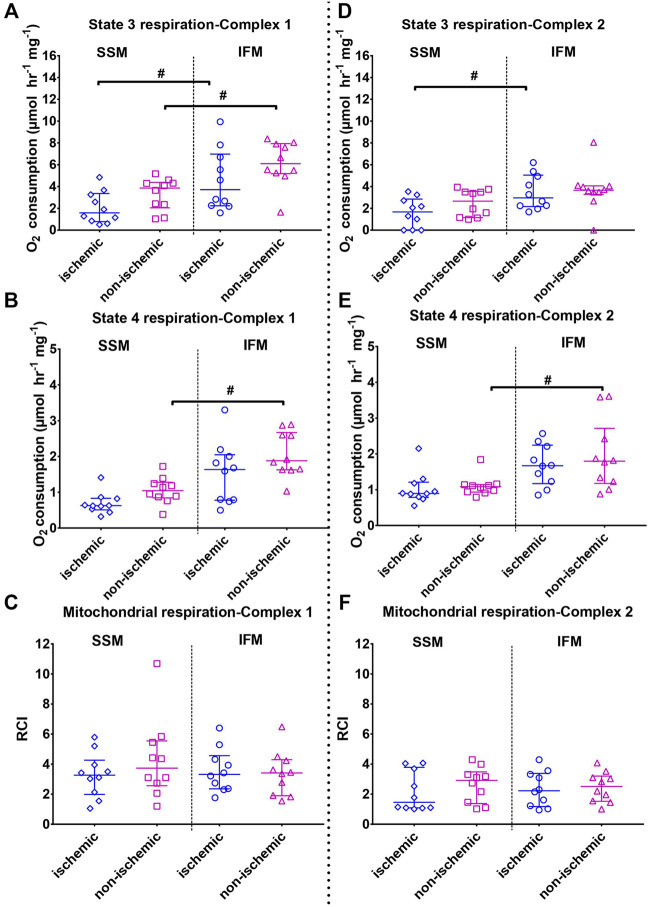
Mitochondrial state 3 (top) and state 4 (middle) oxygen consumptions and the respiratory control indices (RCI, state 3/state 4, bottom panels). Left panels **(A–C)** indicates outcomes (state 3, state 4 and RCI, respectively) using mitochondrial complex I substrates (pyruvate + malate); right panel B (D–F) indicates outcomes (state 3, state 4 and RCI, respectively) using mitochondrial complex II substrate (succinate + rotenone). Values are represented as median with interquartile range (*n* = 7–10/group). # indicates *p* ≤ 0.05 (two-tailed) compared to SSM. For exact *p*-values of all group comparisons, please refer to Table 2.

**FIGURE 4:**
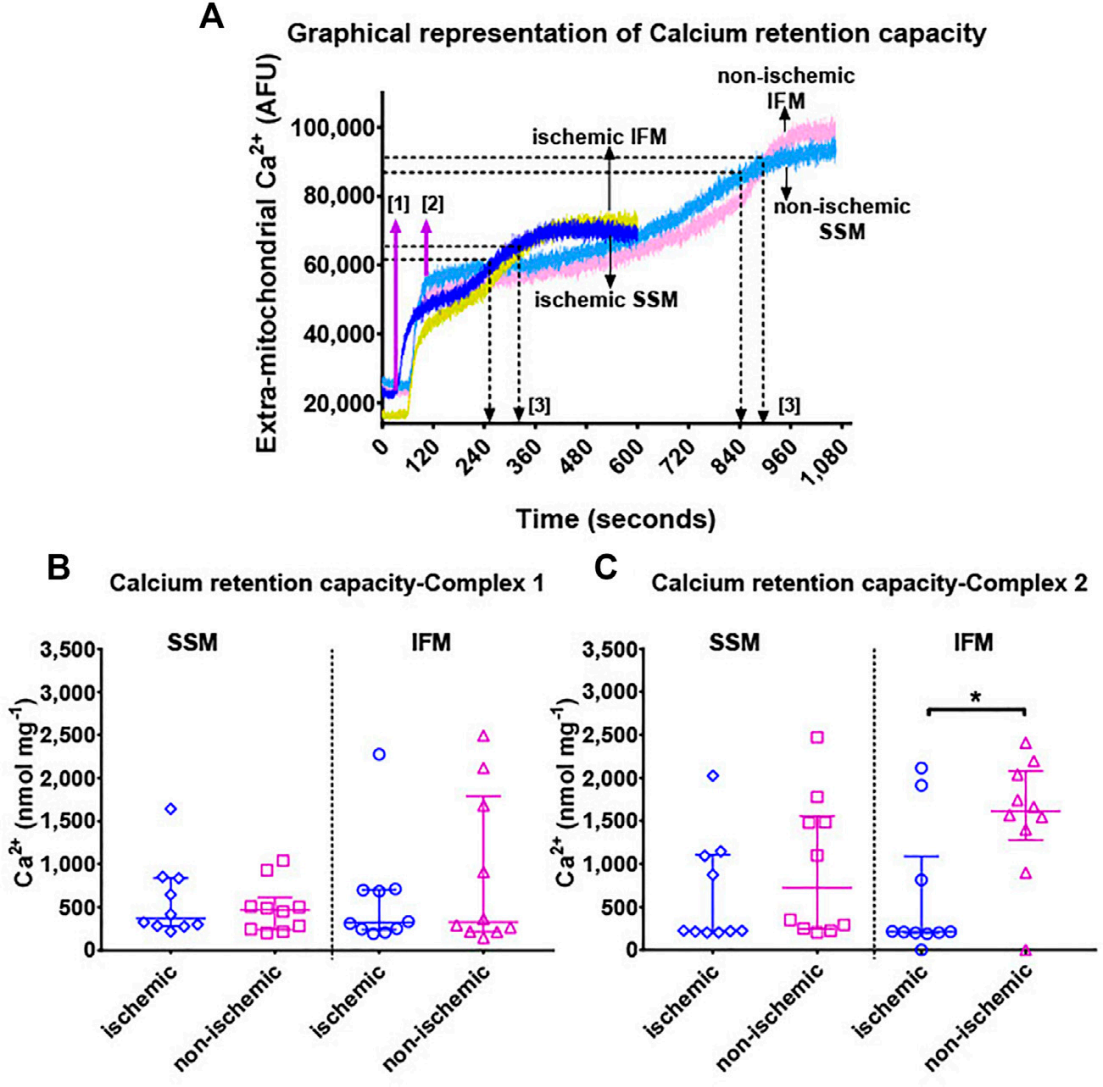
Panel **(A)**: Representative Ca^2+^ retention capacity assay tracings in terms of Ca^2+^ Green fluorescence over time. Continuous infusion of 5 mM Ca^2+^ enters the cuvette over time at the rate of 30 μL min^−1^. Extra-mitochondrial Ca^2+^ (in arbitrary fluorescence units [AFU], indicated on the *y*-axis) is measured by Ca^2+^ Green. At the start of the experiment, assay buffer containing Ca^2+^ Green, mitochondria and substrates are added to the cuvette without any external addition of Ca^2+^. A baseline measurement is taken for about 100 s, after which, a continuous infusion of Ca^2+^ was started using a syringe pump at the rate of 30 μL min^−1^. This is indicated by step [1] in magenta arrows. The addition of Ca^2+^ leads to an immediate spike up until [2], which forms the new baseline for Ca^2+^ levels within the mitochondria. As Ca^2+^ is constantly taken up into the mitochondria at a rate approximately equal to the infusion, extra-mitochondrial Ca^2+^ remains relatively stable. When the maximum calcium retention capacity is reached, mitochondria removes excess Ca^2+^ out of its membrane, and hence the extra-mitochondrial Ca^2+^ signal starts increasing. This increase reaches a plateau when the mPTP opens and all of the mitochondrial Ca^2+^ is released into the cuvette. Black arrows and [3] indicate the time corresponding to the maximum Ca^2+^ concentration that the mitochondria in these particular experiments could tolerate, before the opening of mPTP. This maximum concentration of Ca^2+^ administered over the total duration of infusion yields the total calcium retention capacity. Panels **(B,C)** show mitochondrial calcium retention capacity using complex I (pyruvate + malate) and complex II (succinate + rotenone) substrates, respectively. Values are represented as median with interquartile range (*n* = 9–10/group). * indicates *p* ≤ 0.05 (two-tailed) compared to ischemic tissue. For exact *p*-values of all group comparisons, please refer to Table 2.

**FIGURE 5:**
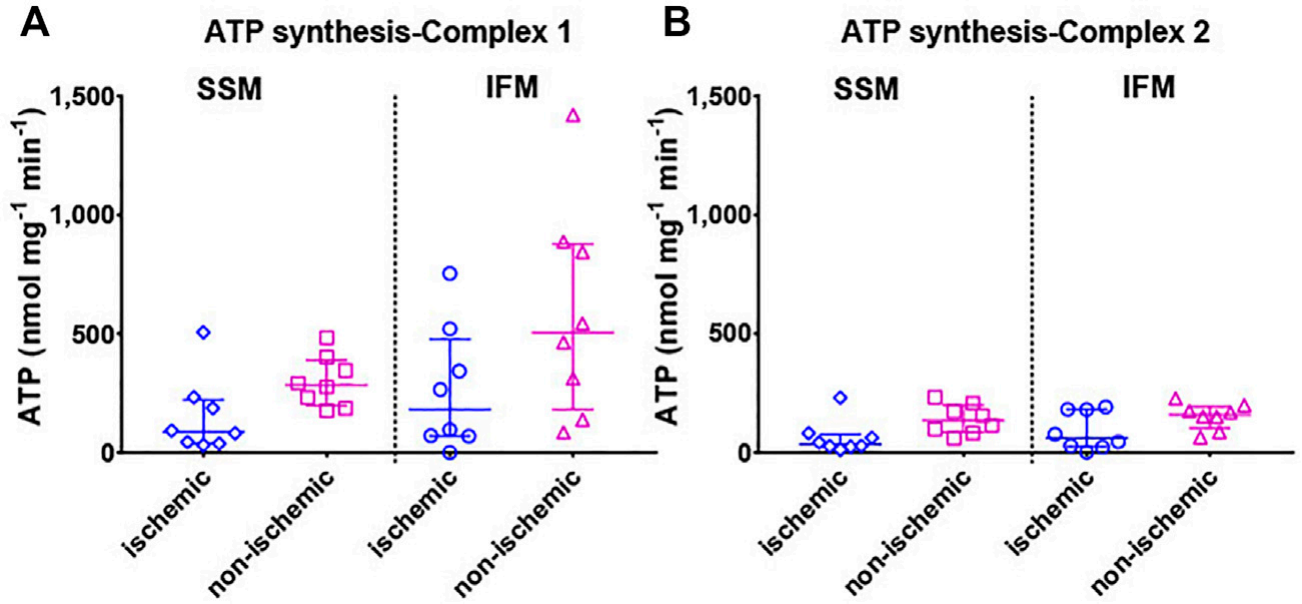
Mitochondrial ATP production rate is shown with **(A)** mitochondrial complex I (pyruvate + malate) and **(B)** complex II (succinate + rotenone) substrates. Values are represented as median with interquartile range (*n* = 7–8/group). For exact *p*-values of all group comparisons, please refer to Table 2.

Our goal was to describe the effects of AMI in the two mitochondrial subpopulations and to compare the function of cardiac SSM and IFM between the ischemic and the non-ischemic area of the heart. For easier reading, no median values or interquartile ranges are stated in the text below, for which the readers are referred to Figures 2–5. Numerical p-values for all the assessments performed between the 207 groups are displayed in Table 2.

**TABLE 2:** *p*-values for overall and all between-group comparisons of mitochondrial function tests between cardiac SSM and IFM within the ischemic (LAD) and non-ischemic (Circ) regions of the saline treated heart displayed in figures 2 to 5. Overall *p*-value was obtained through Friedman’s non-parametric test; pairwise comparison *p*-values were obtained through Dunn’s test.

Mitochondrial function parameter	Overall *p*-value	*p*-values for pairwise comparisons			
		Ischemic v non-ischemic tissue		SSM v IFM	
		SSM	IFM	Ischemic	Non-ischemic
Mitochondrial protein yield (% per g tissue)	0.05*	0.11	0.58	>0.99	>0.99
S3 Respiration Complex I (µmol hr^−1^ mg^−1^)	<0.01*	0.48	0.48	0.04*	0.04*
S4 Respiration Complex I (µmol hr^−1^ mg^−1^)	<0.01*	0.90	0.23	0.10	0.01*
RCI Complex I	0.52	0.90	>0.99	>0.99	0.90
S3 Respiration Complex II (µmol hr^−1^ mg^−1^)	<0.01*	>0.99	>0.99	0.02*	0.10
S4 Respiration Complex II (µmol hr^−1^ mg^−1^)	<0.01*	>0.99	>0.99	0.12	0.03*
RCI Complex II	0.51	0.56	>0.99	>0.99	>0.99
CRC Complex I (nmol mg^−1^)	0.99	>0.99	>0.99	>0.99	>0.99
CRC Complex II (nmol mg^−1^)	0.01*	0.66	<0.01*	>0.99	>0.99
ATP Synthesis Complex I (nmol mg^−1^ min^−1^)	0.18	0.98	0.49	>0.99	>0.99
ATP Synthesis Complex II (nmol mg^−1^ min^−1^)	0.08	0.33	0.70	0.98	>0.99

#### 3.2.1 Mitochondrial Yield

Mitochondrial protein yield was measured using the Bradford assay. Although non-parametric analysis of variance of the yield indicated one or more significant differences among groups, post-hoc comparisons within each region (ischemic or non-ischemic) between SSM and IFM, or within each subpopulation (SSM or IFM) between ischemic and non-ischemic regions did not yield any statistical significance (Figure 2).

#### 3.2.2 Mitochondrial Respiration

Mitochondrial respiration was measured with substrates that target mitochondrial complexes I and II (Figure 3). S3 respiration rates for complex I substrates were significantly reduced in SSM of both the ischemic and the non-ischemic regions, compared to their respective IFMs (overall *p* < 0.01 for both complex I and II substrates). On the other hand, S3 rates of complex II substrate indicated observable differences only for ischemic SSM, compared to ischemic IFM.

S4 respiration rates using complex I and II substrates indicated that in the healthy, non-ischemic region, SSM had a lower state 4 rate than the IFM (overall *p* < 0.01 for both complex I and II substrates). This difference was not observed between the ischemic region SSM and IFM.

No changes with RCI were observed in either subtype with injury (overall *p* = 0.52 for both complex I substrates and *p* = 0.51 for complex II substrate).

#### 3.2.3 Mitochondrial Calcium Retention Capacity

Measurement of total Ca^2+^ the mitochondria can take up and retain (Figure 4A) before opening of the mPTP revealed overall significant differences in CRC using complex II substrate (overall *p* = 0.98 for complex I substrates, and *p* = 0.007 for complex II substrate). However, while performing individual pairwise comparisons (Figure 4, both panels), the only detectable difference was with CRC using complex II substrates, where the ischemic IFM had significantly lower CRC than its non-ischemic counterpart, indicating that in ischemia, Ca^2+^ stress in the IFM leads to early opening of mPTP when compared to the non-ischemic region.

#### 3.2.4 Rate of ATP Production

Mitochondrial ATP production was measured using luminometry (Figure 5). Evaluation of ATP production rates within each territory mimicked the outcomes of RCI in that no statistically significant differences were observed between the ATP production rates of the two mitochondrial subtypes in both the ischemic and the non-ischemic areas (overall *p* = 0.18 for complex I substrate-mediated ATP synthesis and *p* = 0.08 for complex II substrate-mediated ATP synthesis).

## 4 Discussion

In this study, we described the effect of AMI on the two cardiac mitochondrial subpopulations in a clinically relevant large animal model. AMI is commonly caused due to coronary artery disease (Vogel et al., 2019), leading to plaque formation in the arteries supplying blood to the heart. While prolonged ischemia by itself is detrimental, sudden reintroduction of oxygen through reperfusion increases ROS production and stimulation of inflammatory cytokines in the cell, ultimately causing IR injury. Mitochondria play a central role in the signaling events during IR (Murphy and Steenbergen, 2008; Lemieux and Hoppel, 2009; Gazmuri and Radhakrishnan, 2012; Lu et al., 2016).

Cardiac and skeletal muscle mitochondria exist as two functionally distinct subtypes: SSM, below the sarcolemma and IFM between the contractile myofibrils (Palmer et al., 1985; Kuznetsov et al., 2006; Hollander et al., 2014). A third type of mitochondria, known as perinuclear mitochondria (Al-Mehdi et al., 2012; Papatriantafyllou, 2012), have been observed adjacent to the nucleus; these were outside the scope of this current project. Initially thought to differ only by spatial location, researchers have since found that these two subtypes also differ in structure, morphology and biochemical composition (Palmer et al., 1977, 1985). ATP produced by SSM and IFM are believed to be consumed for active protein transport and muscle contraction, respectively. These two subpopulations have varied protein turnover rates (Kasumov et al., 2013) both in normal and pathophysiological states. SSM were shown to be affected in several diseases including ischemia (Shin et al., 1989) and type 2 diabetes (Ritov et al., 2005). IFMs were shown to be preferentially affected in aging (Hofer et al., 2009; Asemu et al., 2013) and pressure overload heart failure (Schwarzer et al., 2013; Shekar et al., 2014). In a rat model of AMI, both SSM and IFM showed poor mitochondrial function (Heather et al., 2010). In an *ex-vivo* ischemic heart model in rabbits (Lesnefsky et al., 1997), SSM were indicated to have reduced oxidative phosphorylation with ischemia. In a related study, this reduction in ischemia-induced oxidative phosphorylation was attributed to the partial depletion in the cardiolipin content (Croston et al., 2013) specifically in the SSM, while the IFM remained relatively unaffected. Moreover, several therapies (Kajiyama et al., 1987; Duan and Karmazyn, 1989; Ovize et al., 2010; Vergeade et al., 2010; Holmuhamedov et al., 2012; Lesnefsky et al., 2017) have also shown to preferentially improve the function of one mitochondrial subtype over the other. There have been a few studies that investigated the effect of IR in large animal models, either by directly targeting the damaged tissue or by utilizing mitochondria-targeted therapeutic interventions to treat reperfusion injury (Kajiyama et al., 1987; Ide et al., 2019; Shin et al., 2019; Traverse et al., 2019); however, our study is unique in employing the STEMI model of AMI, which is one of the most severe forms of coronary artery disease in humans, with a different manifestation of ischemia as opposed to frequently employed circumflex artery occlusion or permanent ligation of the left anterior descending artery, which would be indicative of a chronic model of heart failure. Furthermore, the 4-h reperfusion timepoint allows for the infarction to develop and detection of biomarkers in the blood, as opposed to immediate harvest of the heart at reperfusion, which has been described in our prior work. To our knowledge, there has been no large animal model of AMI where the roles of SSM and IFM were examined in the initial few hours of reperfusion.

To confirm if STEMI was specific to the ischemic area, we performed several checks during the study, including assessing blood flow in the LAD artery during ischemia and reperfusion by fluoroscopic imaging. Based on our previous work (Bartos et al., 2016), we chose to study the 4-h reperfusion time point, since we have shown evidence of myocardial damage and the detection of cardiac injury markers in the blood at this time.

When comparing the mitochondrial functions of cardiac SSM and IFM within each affected area, we found no differences in mitochondrial yield or RCI. However, when examining individual states of oxygen consumption using both complex I and complex II substrates, ischemic SSM had markedly reduced S3 respiration rate compared to ischemic IFM. In the non-ischemic territory, this difference was found only with complex I substrates. With state 4, both complex I and II substrates showed that in the non-ischemic region, IFM had higher S4 than the SSM as observed in similar works (Croston et al., 2013; Ertracht et al., 2014). This difference disappeared with ischemia, thereby indicating a trend towards reduction in IFM S4 levels with ischemia. This demonstrates a preferential effect with the electron transport chain complex function with this mitochondrial subtype. We did not evaluate the individual enzyme activities for any of the electron transport chain (ETC) complexes in this study based on the already well-established literature related to the levels of these enzymes in reperfusion injury. An indirect assessment of oxidative stress was performed by measuring the calcium retention capacity of the mitochondria, since excessive ROS generation is one of the main triggers of the opening of mPTP for the release of calcium from the mitochondria (Seidlmayer et al., 2015). CRC remained unaffected with both SSM and IFM with complex I substrates while it was significantly reduced in ischemic IFM with complex II substrates compared to their non-ischemic IFM; this points to a potential ETC complex II-mediated dysfunction related to CRC *In toto*, no consistent differences were found between SSM and IFM with regards to CRC or ATP production, indicating that the major distinction between these two subtypes in a large animal model is in oxygen consumption.

One of the unique aspects of this work is that few studies (Heather et al., 2010) have compared the role of cardiac SSM and IFM immediately after an AMI, but none in a large animal model. The existing literature has described the effects of prolonged AMI or heart failure (Rosca et al., 2011; O’Connell et al., 2013; Shekar et al., 2014), which happen days to weeks after the ischemic event. Several reactions including Ca^2+^ overload and recruitment of infiltrating immune cells including neutrophils and macrophages happen early on reperfusion, thus providing a rationale for our 4-h reperfusion period before organ harvest. This pathophysiological adaptation of mitochondria is instrumental in shaping the long-term compensatory response that helps in myocardial remodeling. Of note, is that this study was designed as the foundation to better understand the subcellular mechanisms that occur in a pre-clinical AMI model. These findings are essential to design future studies that investigate the cellular mechanisms behind the heterogeneity in mitochondrial function between cardiac SSM and IFM during AMI and how cardioprotective therapies might affect each subpopulation.

## 5 Limitations

Our analysis on the effect of injury after 45 min of ischemia and 4 h of reperfusion indicated some significant differences overall in mitochondrial function outcomes, while at times failing to detect consistent pairwise differences with a *p* value of 0.05 or less. Another notable limitation of this work is that we observed a significant data spread owing to variation among the animals used in this study. Since the pigs were not bred in a controlled manner, we were unable to maintain the same genomic homogeneity typically observed in rodent studies. An increase in the sample size to increase power and avoid a possible type II error could be a solution but substantial costs associated with performing these labor-intense porcine experiments prohibit us from doing so at this time. Moreover, we cannot exclude the possibility that a smaller proportion of the mitochondria from the ischemic area were obtained from dead tissue or could have undergone fragmentation due to the isolation process. Likewise, we did not measure the levels of cardiac and mitochondrial enzymes including the ETC component activities, which potentially could have provided a mechanistic explanation and possibly strengthened the results obtained. Despite the logistical challenges in conducting several concurrent experiments, the primary goal of this work was to provide a descriptive analysis on the immediate effects on the two mitochondrial subpopulations post AMI. The mechanistic connection between differential mitochondrial effect on AMI and enzyme activities is within the scope of subsequent work.

Previously, we utilized a 2,3,5-Triphenyltetrazolium chloride and Evan’s blue based staining method (Bartos et al., 2016) to differentiate between the area-at-risk and dead tissue; however, we were unable to do so in this case due to the possibility of the stains interfering with mitochondrial function. Our study also does not include a complete naïve subgroup of animals without a STEMI; to avoid using additional animals for this group, we chose to use the remotely affected circumflex artery area from each animal. We performed sham surgeries to a small cohort of animals in a separate unpublished study and did not find any differences in mitochondrial function between the LAD and the circumflex artery area. Additionally, in our model of IR, the use of isoflurane during the entire procedure is warranted to ensure animal welfare and anesthesia; however, several studies in the past have indicated that volatile anesthetics including isoflurane (Qian et al., 2005; Pravdic et al., 2010; Lotz et al., 2015) and sevoflurane (Riess et al., 2002, 2003, 2014; Bartos et al., 2015) have protective effects on the myocardium as well as the mitochondria. Nevertheless, all test animals received comparable concentrations of isoflurane for the same duration of time and, hence, any change in mitochondrial function was independent of the effect of isoflurane used during the study. Of note is that the method of statistical analyses used in this study was based on a conservative approach, assuming non-normal distribution. Even though more rigorous, this method of analysis has a higher chance of Type II errors so that pairwise differences were not always significant. Finally, we used all female pigs (3–4 months old, pre-pubescent) for this experiment to mimic the animal model from our previous study (Bartos et al., 2016) and to reduce confounding variables. Currently, the role of gender in cardiac mitochondrial subpopulations is not fully known. While in some animal models, female hearts seem less susceptible to reperfusion injury (Bae and Zhang, 2005; Johnson et al., 2006; Ostadal et al., 2009), there are no strong clinical indications (Maznyczka et al., 2019) to support this conclusion, and the results are often inconclusive (McCully et al., 2006). Hence, our current results are valid for female, but not necessarily male pigs.

## 6 Conclusion

In conclusion, we present a large animal model of AMI where SSM and IFM have different susceptibilities to IR damage, based on various mitochondrial function tests. We observed that in our clinically relevant acute reperfusion model, ischemic SSM had reduced S3 respiration compared to the ischemic IFM**.** Despite detecting overall differences with reperfusion, we did not find any consistent differences in mitochondrial functional assessments either between mitochondrial subtypes or between ischemic and non-ischemic zones, hence indicating that both mitochondrial subtypes undergo significant dysfunction in the initial 4 h of reperfusion injury.

## Data Availability

The original contributions presented in the study are included in the article, further inquiries can be directed to the corresponding author.
